# On the Intended Surgeons' Bill, as Far as It Relates to Midwifery

**Published:** 1816-10

**Authors:** John Walker

**Affiliations:** Director of the Royal Jennerian and London Vaccine Institutions.


*For the London Medical and Physical Journal.*

*On the intended Surgeons' Bill, as far as it relates to Midwifery;* by John Walker, M.D. Director of the Royal Jennerian and London Vaccine Institutions.

OF all the parts of the medical profession, or practice, that of the accoucheur must be considered the most sacred,—the least fit to be subjected to legislative meddlings or enactments. Yet, in the Surgeons' Bill, intended to be introduced into Parliament in the next session, it is proposed to be enacted,—"Whereas surgical aid is frequently required in midwifery, and it is expedient that male persons so practising should be qualified to render such aid, that it shall not be lawful for any male person to practise midwifery, unless he shall have obtained a diploma, or testimonial of his knowledge and ability, to practise surgery, under the seal of one of the said three Colleges (London, Dublin, Edinburgh), or unless he shall have obtained a testimonial of qualification as a principal surgeon in the army or navy, and shall actually have served in that capacity," &c. &c.

The Royal College of Physicians once assumed the authority of granting their permission or licence to practise midwifery. What has been the consequence of such assumption? The vain attempt of the learned body to establish their authority over a practice in which hardly one of them could pretend, would even design to pretend, he had seen service, proved perfectly nugatory. Their order of Licentiates of Midwifery is nearly extinct. The passing of the surgeons' bill would, now, so completely extinguish the assumed authority, that even the members of their College would not be permitted to practise midwifery, because surgical assistance is frequently required, and the fellows and the licentiates, in their admission into the College of Physicians, are obliged to prove that they are no surgeons.

What is to become of the general practitioner, the regular apothecary, now legislatively recognised as a practitioner, if the bill be allowed to pass in its present form? In vain will his

 friend, friend, his neighbour, in the heart-comforting confidence which he feels in the whole character of the worthy practitioner, address him on the most important concern of domestic life. During the anxious period of gestation, his seasonable visits may often gladden the family; and, consoled by his counsel, they may look forwards with cheering hope to the eventful season of parturition. But then at last their hopes must be blighted. All their expectations of his continued solicitude and professional aid will be completely disappointed, for he will then have to declare, while their every hope and confidence may be reposed on him alone, that they must call in a surgeon; that he is by law precluded from rendering his professional services to the alarmed, the agitated patient, and consolation or confidence to her astonished, disappointed, distracted companions; that, whatever symptom of puerperal fever, or other malady, however dire, threatening or present, may appear, neither he nor the physician are eligible to attend the labour. The surgeon only is competent. The law has determined the question; for surgical assistance is frequently required.

*Bond-court, Walbrook;*
13, viii. 1816.

P.S.—The examiners of the Apothecaries' Company examine their candidates for appointment on the subject of midwifery; the similar boards of the Colleges do not. If the surgeons do obtain from Parliament the exclusive privilege of becoming men-midwives, and are not constrained, by the same authority, to practise as such, it may be said that Parliament has inadvertently done great injustice to his Majesty's subjects, by preventing physicians and apothecaries from following a most important practice, which is almost universally declined by the pure surgeon.

---

☞ We think the above remarks worth the attention of the general practitioner, who, after an examination at Apothecaries' Hall, conceives himself authorised to practise midwifery, without which it is in vain to expect employment in the country, and not very easy in the metropolis.—Edit.

